# Supplementary material for: Genome-Wide Association Studies for Sex Determination and Cross-Compatibility in Water Yam (Dioscorea alata L.)
Source: Plants (Basel). 2021 Jul 10;10(7):1412. doi: 10.3390/plants10071412 (PMC8309230; doi:10.3390/plants10071412)
Supplement: Supplementary file 1 [file plants-10-01412-s001.zip › References for Table S3.pdf]

References for supplementary Table S3

1. Arnaud, N. and Pautot, V., 2014. Ring the BELL and tie the KNOX: roles for TALEs in gynoecium development. *Frontiers in Plant Science*, *5*, p.93.
2. Asano, T., Tanaka, N., Yang, G., Hayashi, N. and Komatsu, S., 2005. Genome-wide identification of the rice calcium-dependent protein kinase and its closely related kinase gene families: comprehensive analysis of the CDPKs gene family in rice. *Plant and Cell Physiology*, *46*(2), pp.356–366.
3. Aso, K., Kato, M., Banks, J.A. and Hasebe, M., 1999. Characterization of homeodomain-leucine zipper genes in the fern *Ceratopteris richardii* and the evolution of the homeodomain-leucine zipper gene family in vascular plants. *Molecular Biology and Evolution*, *16*(4), pp.544–552.
4. Baloglu, M.C., Eldem, V., Hajyzadeh, M. and Unver, T., 2014. Genome-wide analysis of the bZIP transcription factors in cucumber. *PloS one*, *9*(4), p.e96014.
5. Bararyenya, A., Olukolu, B.A., Tukamuhabwa, P., Grüneberg, W.J., Ekaya, W., Low, J., Ochwo-Ssemakula, M., Odong, T.L., Talwana, H., Badji, A. and Kyalo, M., 2020. Genome-wide association study identified candidate genes controlling continuous storage root formation and bulking in hexaploid sweetpotato. *BMC Plant Biology*, *20*(1), pp.1–16.
6. Book, A.J., Smalle, J., Lee, K.H., Yang, P., Walker, J.M., Casper, S., Holmes, J.H., Russo, L.A., Buzzinotti, Z.W., Jenik, P.D. and Vierstra, R.D., 2009. The RPN5 subunit of the 26S proteasome is essential for gametogenesis, sporophyte development, and complex assembly in Arabidopsis. *The Plant Cell*, *21*(2), pp.460–478.
7. Bortiri, E. and Hake, S., 2007. Flowering and determinacy in maize. *Journal of Experimental Botany*, *58*(5), pp.909–916.
8. Carmichael, S.N., Bekaert, M., Taggart, J.B., Christie, H.R., Bassett, D.I., Bron, J.E., Skuce, P.J., Gharbi, K., Skern-Mauritzen, R. and Sturm, A., 2013. Identification of a sex-linked SNP marker in the salmon louse (*Lepeophtheirus salmonis*) using RAD sequencing. *Plos one*, *8*(10), p.e77832.
9. Chae, T., 2018. Sex-linked differential gene expression in *Carica papaya*. Doctoral dissertation, Miami University, USA.
10. Cheng, X., Peng, J., Ma, J., Tang, Y., Chen, R., Mysore, K.S. and Wen, J., 2012. NO APICAL MERISTEM (MtNAM) regulates floral organ identity and lateral organ separation in *Medicago truncatula*. *New Phytologist*, *195*(1), pp.71–84.
11. Costanzo, E., Trehin, C. and Vandenbussche, M., 2014. The role of WOX genes in flower development. *Annals of Botany*, *114*(7), pp.1545–1553.
12. Cucinotta, M., Di Marzo, M., Guazzotti, A., de Folter, S., Kater, M.M. and Colombo, L., 2020. Gynoecium size and ovule number are interconnected traits that impact seed yield. *Journal of Experimental Botany*, *71*(9), pp.2479–2489.
13. Cui, X., Lu, F., Li, Y., Xue, Y., Kang, Y., Zhang, S., Qiu, Q., Cui, X., Zheng, S., Liu, B. and Xu, X., 2013. Ubiquitin-specific proteases UBP12 and UBP13 act in circadian clock and photoperiodic flowering regulation in Arabidopsis. *Plant Physiology*, *162*(2), pp.897–906.
14. Devani, R.S., Chirmade, T., Sinha, S., Bendahmane, A., Dholakia, B.B., Banerjee, A.K. and Banerjee, J., 2019. Flower bud proteome reveals modulation of sex-biased proteins potentially associated with sex expression and modification in dioecious *Coccinia grandis*. *BMC Plant Biology*, *19*(1), pp.1–15.
15. Dong, X.; Jiang, Y.; Hur, Y. Genome-Wide Analysis of Glycoside Hydrolase Family 1 β-glucosidase Genes in *Brassica rapa* and Their Potential Role in Pollen Development. *Int. J. Mol. Sci.* 2019, 20, 1663. https://doi.org/10.3390/ijms20071663
16. Eleblu, J.S., Haraghi, A., Mania, B., Camps, C., Rashid, D., Morin, H., Dogimont, C., Boualem, A. and Bendahmane, A., 2019. The gynoecious CmWIP1 transcription factor interacts with CmbZIP48 to inhibit carpel development. *Scientific Reports*, *9*(1), pp.1–10.
17. Erickson, R.P. and Verga, V., 1989. Is zinc-finger Y the sex-determining gene?. *American Journal of Human Genetics*, *45*(5), p.671.
18. Fang, C., Ma, Y., Wu, S., Liu, Z., Wang, Z., Yang, R., Hu, G., Zhou, Z., Yu, H., Zhang, M. and Pan, Y., 2017. Genome-wide association studies dissect the genetic networks underlying agronomical traits in soybean. *Genome Biology*, *18*(1), pp.1–14.
19. Footitt, S., Dietrich, D., Fait, A., Fernie, A.R., Holdsworth, M.J., Baker, A. and Theodoulou, F.L., 2007. The COMATOSE ATP-binding cassette transporter is required for full fertility in Arabidopsis. *Plant Physiology*, *144*(3), pp.1467–1480.
20. Gaborieau, L., Brown, G.G. and Mireau, H., 2016. The propensity of pentatricopeptide repeat genes to evolve into restorers of cytoplasmic male sterility. *Frontiers in Plant Science*, *7*, p.1816.
21. Gachomo, E.W., Jimenez-Lopez, J.C., Baptiste, L.J. and Kotchoni, S.O., 2014. GIGANTUS1 (GTS1), a member of Transducin/WD40 protein superfamily, controls seed germination, growth and biomass accumulation through ribosome-biogenesis protein interactions in *Arabidopsis thaliana*. *BMC Plant Biology*, *14*(1), pp.1–17.

22. Gady, A.L., Alves, C.S. and Nogueira, F.T., 2017. Epigenetics in plant reproductive development: an overview from flowers to seeds. *Plant Epigenetics*, pp.329–357.
23. García, A., Aguado, E., Garrido, D., Martínez, C. and Jamilena, M., 2020. Two androecious mutations reveal the crucial role of ethylene receptors in the initiation of female flower development in *Cucurbita pepo*. *The Plant Journal*, *103*(4), pp.1548–1560.
24. García, A., Aguado, E., Martínez, C., Loska, D., Beltrán, S., Valenzuela, J.L., Garrido, D. and Jamilena, M., 2020. The ethylene receptors CpETR1A and CpETR2B cooperate in the control of sex determination in *Cucurbita pepo*. *Journal of Experimental Botany*, *71*(1), pp.154–167.
25. Ghadge, A.G., Karmakar, K., Devani, R.S., Banerjee, J., Mohanasundaram, B., Sinha, R.K., Sinha, S. and Banerjee, A.K., 2014. Flower development, pollen fertility and sex expression analyses of three sexual phenotypes of *Coccinia grandis*. *BMC Plant Biology*, *14*(1), pp.1–15.
26. Girma, G., Natsume, S., Carluccio, A.V., Takagi, H., Matsumura, H., Uemura, A., Muranaka, S., Takagi, H., Stavolone, L., Gedil, M. and Spillane, C., 2019. Identification of candidate flowering and sex genes in white Guinea yam (D. rotundata Poir.) by SuperSAGE transcriptome profiling. *PloS One*, *14*(9), p.e0216912.
27. Graham, P.L., Yanowitz, J.L., Penn, J.K., Deshpande, G. and Schedl, P., 2011. The translation initiation factor eIF4E regulates the sex-specific expression of the master switch gene Sxl in *Drosophila melanogaster*. *PLoS Genet*, *7*(7), p.e1002185.
28. Guan, Y., Ding, L., Jiang, J., Shentu, Y., Zhao, W., Zhao, K., Zhang, X., Song, A., Chen, S. and Chen, F., 2021. Overexpression of the CmJAZ1-like gene delays flowering in *Chrysanthemum morifolium*. *Horticulture Research*, *8*(1), pp.1–12.
29. Gutsche, N. and Zachgo, S., 2016. The N-terminus of the floral Arabidopsis TGA transcription factor PERIANTHIA mediates redox-sensitive DNA-binding. *PLoS One*, *11*(4), p.e0153810.
30. Hamès, C., Ptchelkine, D., Grimm, C., Thevenon, E., Moyroud, E., Gérard, F., Martiel, J.L., Benlloch, R., Parcy, F. and Müller, C.W., 2008. Structural basis for LEAFY floral switch function and similarity with helix-turn-helix proteins. *The EMBO Journal*, *27*(19), pp.2628–2637.
31. Hardenack, S., Ye, D., Saedler, H. and Grant, S., 1994. Comparison of MADS box gene expression in developing male and female flowers of the dioecious plant white campion. *The Plant Cell*, *6*(12), pp.1775–1787.
32. Ito, S., Song, Y.H., Josephson-Day, A.R., Miller, R.J., Breton, G., Olmstead, R.G. and Imaizumi, T., 2012. FLOWERING BHLH transcriptional activators control expression of the photoperiodic flowering regulator CONSTANS in Arabidopsis. *Proceedings of the National Academy of Sciences*, *109*(9), pp.3582–3587.
33. Jones, S., 2004. An overview of the basic helix-loop-helix proteins. *Genome Biology*, *5*(6), pp.1–6.
34. Kater, M.M., Franken, J., Carney, K.J., Colombo, L. and Angenent, G.C., 2001. Sex determination in the monoecious species cucumber is confined to specific floral whorls. *The Plant Cell*, *13*(3), pp.481–493.
35. Khadka, J., Yadav, N.S., Guy, M., Grafi, G. and Golan-Goldhirsh, A., 2019. Epigenetic aspects of floral homeotic genes in relation to sexual dimorphism in the dioecious plant *Mercurialis annua*. *Journal of Experimental Botany*, *70*(21), pp.6245–6259.
36. Kohno, S., Katsu, Y., Urushitani, H., Ohta, Y., Iguchi, T. and Guillette Jr, L.J., 2010. Potential contributions of heat shock proteins to temperature-dependent sex determination in the American alligator. *Sexual Development*, *4*(1-2), pp.73–87.
37. Li, D., Sheng, Y., Niu, H. and Li, Z., 2019. Gene interactions regulating sex determination in cucurbits. *Frontiers in Plant Science*, *10*, p.1231.
38. Li, H.Y. and Gray, J.E., 1997. Pollination-enhanced expression of a receptor-like protein kinase related gene in tobacco styles. *Plant Molecular Biology*, *33*(4), pp.653–665.
39. Li, Q. and Liu, B., 2017. Genetic regulation of maize flower development and sex determination. *Planta*, *245*(1), pp.1–14.
40. Li, S.F., Zhang, G.J., Zhang, X.J., Yuan, J.H., Deng, C.L. and Gao, W.J., 2017. Comparative transcriptome analysis reveals differentially expressed genes associated with sex expression in garden asparagus (*Asparagus officinalis*). *BMC Plant Biology*, *17*(1), pp.1–16.
41. Lin, Z., Ho, C.W. and Grierson, D., 2009. AtTRP1 encodes a novel TPR protein that interacts with the ethylene receptor ERS1 and modulates development in Arabidopsis. *Journal of Experimental Botany*, *60*(13), pp.3697–3714.
42. Liu, J., Cheng, X., Liu, P., Li, D., Chen, T., Gu, X. and Sun, J., 2017. MicroRNA319-regulated TCPs interact with FBHs and PFT1 to activate CO transcription and control flowering time in Arabidopsis. *PLoS Genetics*, *13*(5), p.e1006833.
43. Luo, Y., Pan, B.Z., Li, L., Yang, C.X. and Xu, Z.F., 2020. Developmental basis for flower sex determination and effects of cytokinin on sex determination in *Plukenetia volubilis* (Euphorbiaceae). *Plant Reproduction*, pp.1–14.

44. Lv, X., Lan, S., Guy, K.M., Yang, J., Zhang, M. and Hu, Z., 2016. Global expressions landscape of NAC transcription factor family and their responses to abiotic stresses in *Citrullus lanatus*. *Scientific Reports*, *6*(1), pp.1–14.
45. Maier, A.T., Stehling-Sun, S., Wollmann, H., Demar, M., Hong, R.L., Haubeiß, S., Weigel, D. and Lohmann, J.U., 2009. Dual roles of the bZIP transcription factor PERIANTHIA in the control of floral architecture and homeotic gene expression. *Development*, *136*(10), pp.1613–1620.
46. Mao, Y., Liu, W., Chen, X., Xu, Y., Lu, W., Hou, J., Ni, J., Wang, Y. and Wu, L., 2017. Flower development and sex determination between male and female flowers in *Vernicia fordii*. *Frontiers in Plant Science*, *8*, p.1291.
47. Massonnet, M., Cochetel, N., Minio, A., Vondras, A.M., Lin, J., Muyle, A., Garcia, J.F., Zhou, Y., Delledonne, M., Riaz, S. and Figueroa-Balderas, R., 2020. The genetic basis of sex determination in grapes. *Nature Communications*, *11*(1), pp.1–12.
48. Matsuhira, H., Kagami, H., Kurata, M., Kitazaki, K., Matsunaga, M., Hamaguchi, Y., Hagihara, E., Ueda, M., Harada, M., Muramatsu, A. and Yui-Kurino, R., 2012. Unusual and typical features of a novel restorer-of-fertility gene of sugar beet (*Beta vulgaris* L.). *Genetics*, *192*(4), pp.1347–1358.
49. Matsuhisa, S. and Ushimaru, A., 2015. Sexual dimorphism in floral longevity and flowering synchrony in relation to pollination and mating success in three dioecious Ilex species. *American Journal of Botany*, *102*(7), pp.1187–1197.
50. Michelini, S., Chiurazzi, P., Marino, V., Dell'Orco, D., Manara, E., Baglivo, M., Fiorentino, A., Maltese, P.E., Pinelli, M., Herbst, K.L. and Dautaj, A., 2020. Aldo-Keto Reductase 1C1 (AKR1C1) as the First Mutated Gene in a Family with Nonsyndromic Primary Lipedema. *International Journal of Molecular Sciences*, *21*(17), p.6264.
51. Montalvão, A.P.L., Kersten, B., Fladung, M. and Müller, N.A., 2020. The Diversity and Dynamics of Sex Determination in Dioecious Plants. *Frontiers in Plant Science*, *11*.
52. Mukherjee, K., Brocchieri, L. and Bürglin, T.R., 2009. A comprehensive classification and evolutionary analysis of plant homeobox genes. Molecular biology and evolution, 26(12), pp.2775-2794.
53. Murase, K., Shigenobu, S., Fujii, S., Ueda, K., Murata, T., Sakamoto, A., Wada, Y., Yamaguchi, K., Osakabe, Y., Osakabe, K. and Kanno, A., 2017. MYB transcription factor gene involved in sex determination in *Asparagus officinalis*. *Genes to Cells*, *22*(1), pp.115–123.
54. Nagai, J.I., Yamato, K.T., Sakaida, M., Yoda, H., Fukuzawa, H. and Ohyama, K., 1999. Expressed sequence tags from immature female sexual organ of a liverwort, *Marchantia polymorpha*. *DNA Research*, *6*(1), pp.1–11.
55. Nicolas, M. and Cubas, P., 2016. The role of TCP transcription factors in shaping flower structure, leaf morphology, and plant architecture. In *Plant Transcription Factors* (pp. 249–267). Academic Press.
56. Park, B.S., Eo, H.J., Jang, I.C., Kang, H.G., Song, J.T. and Seo, H.S., 2010. Ubiquitination of LHY by SINAT5 regulates flowering time and is inhibited by DET1. *Biochemical and Biophysical Research Communications*, *398*(2), pp.242–246.
57. Patil, R.V. and Pawar, K.D., 2019. Comparative de novo flower transcriptome analysis of polygamodioecious tree *Garcinia indica*. *3 Biotech*, *9*(3), pp.1–15.
58. Pawełkowicz, M., Pryszcz, L., Skarzyńska, A., Wóycicki, R.K., Posyniak, K., Rymuszka, J., Przybecki, Z. and Pląder, W., 2019. Comparative transcriptome analysis reveals new molecular pathways for cucumber genes related to sex determination. *Plant Reproduction*, *32*(2), pp.193–216.
59. Petit, J., Salentijn, E.M., Paulo, M.J., Denneboom, C. and Trindade, L.M., 2020. Genetic Architecture of Flowering Time and Sex Determination in Hemp (*Cannabis sativa* L.): A Genome-Wide Association Study. *Frontiers in Plant Science*, *11*, p.1704.
60. Picq, S., Santoni, S., Lacombe, T., Latreille, M., Weber, A., Ardisson, M., Ivorra, S., Maghradze, D., Arroyo-Garcia, R., Chatelet, P. and This, P., 2014. A small XY chromosomal region explains sex determination in wild dioecious *V. vinifera* and the reversal to hermaphroditism in domesticated grapevines. *BMC Plant Biology*, *14*(1), pp.1–17.
61. Pipatchartlearnwong, K., Juntawong, P., Wonnapinij, P., Apisitwanich, S. and Vuttipongchaikij, S., 2019. Towards sex identification of Asian Palmyra palm (*Borassus flabellifer* L.) by DNA fingerprinting, suppression subtractive hybridization and de novo transcriptome sequencing. *PeerJ*, *7*, p.e7268.
62. Radkova, M., Revalska, M., Kertikova, D. and Iantcheva, A., 2019. Zinc finger CCHC-type protein related with seed size in model legume species *Medicago truncatula*. *Biotechnology & Biotechnological Equipment*, 33(1), pp.278–285.
63. Raman, H., Raman, R., Qiu, Y., Yadav, A.S., Sureshkumar, S., Borg, L., Rohan, M., Wheeler, D., Owen, O., Menz, I. and Balasubramanian, S., 2019. GWAS hints at pleiotropic roles for FLOWERING LOCUS T in flowering time and yield-related traits in canola. *BMC Genomics*, *20*(1), pp.1–18.
64. Sakuma, S., Golan, G., Guo, Z., Ogawa, T., Tagiri, A., Sugimoto, K., Bernhardt, N., Brassac, J., Mascher, M., Hensel, G. and Ohnishi, S., 2019. Unleashing floret fertility in wheat through the mutation of a homeobox gene. *Proceedings of the National Academy of Sciences*, *116*(11), pp.5182–5187.

65. Sardos, J., Rouard, M., Hueber, Y., Cenci, A., Hyma, K.E., Van Den Houwe, I., Hribova, E., Courtois, B. and Roux, N., 2016. A genome-wide association study on the seedless phenotype in banana (*Musa* spp.) reveals the potential of a selected panel to detect candidate genes in a vegetatively propagated crop. *PLoS One*, *11*(5), p.e0154448.
66. Sharma, N., Xin, R., Kim, D.H., Sung, S., Lange, T. and Huq, E., 2016. NO FLOWERING IN SHORT DAY (NFL) is a bHLH transcription factor that promotes flowering specifically under short-day conditions in Arabidopsis. *Development*, *143*(4), pp.682–690.
67. Shi, T., Gao, Z., Wang, L., Zhang, Z., Zhuang, W., Sun, H. and Zhong, W., 2012. Identification of differentially-expressed genes associated with pistil abortion in Japanese apricot by genome-wide transcriptional analysis. *PloS One*, *7*(10), p.e47810.
68. Shchennikova, A.V., Shulga, O.A., Kochieva, E.Z., Beletsky, A.V., Filyushin, M.A., Ravin, N.V. and Skryabin, K.G., 2017. Homeobox genes encoding WOX transcription factors in the flowering parasitic plant *Monotropa hypopitys*. *Russian Journal of Genetics: Applied Research*, *7*(7), pp.781–788.
69. Shen, L., Kang, Y.G.G., Liu, L. and Yu, H., 2011. The J-domain protein J3 mediates the integration of flowering signals in *Arabidopsis*. *The Plant Cell*, *23*(2), pp.499–514.
70. Singh, S.K., Kumar, V., Srinivasan, R., Ahuja, P.S., Bhat, S.R. and Sreenivasulu, Y., 2017. The TRAF Mediated Gametogenesis Progression (TRAMGaP) gene is required for megaspore mother cell specification and gametophyte development. *Plant physiology*, *175*(3), pp.1220–1237.
71. Skinner, M.K., Rawls, A., Wilson-Rawls, J. and Roalson, E.H., 2010. Basic helix-loop-helix transcription factor gene family phylogenetics and nomenclature. *Differentiation*, *80*(1), pp.1–8.
72. Song, Y., Ma, K., Bo, W., Zhang, Z. and Zhang, D., 2012. Sex-specific DNA methylation and gene expression in andromonoecious poplar. *Plant Cell Reports*, *31*(8), pp.1393–1405.
73. Suzuki, M.G., Imanishi, S., Dohmae, N., Asanuma, M. and Matsumoto, S., 2010. Identification of a male-specific RNA binding protein that regulates sex-specific splicing of Bmdsx by increasing RNA binding activity of BmPSI. *Molecular and Cellular Biology*, *30*(24), pp.5776–5786.
74. Tang, P., Zhang, Q. and Yao, X., 2017. Comparative transcript profiling explores differentially expressed genes associated with sexual phenotype in kiwifruit. *Plos One*, *12*(7), p.e0180542.
75. Tran, F., Penniket, C., Patel, R.V., Provart, N.J., Laroche, A., Rowland, O. and Robert, L.S., 2013. Developmental transcriptional profiling reveals key insights into Triticeae reproductive development. *The Plant Journal*, *74*(6), pp.971–988.
76. Ventura, J., 2012. Characterization of maize sex-determination gene orthologs in rice (*Oryza sativa* L. *japonica* cv. Nipponbare).
77. Wang, L., Yin, H., Qian, Q., Yang, J., Huang, C., Hu, X. and Luo, D., 2009. NECK LEAF 1, a GATA type transcription factor, modulates organogenesis by regulating the expression of multiple regulatory genes during reproductive development in rice. *Cell Research*, *19*(5), pp.598–611.
78. Wang, W. and Zhang, X., 2017. Identification of the sex-biased gene expression and putative sex-associated genes in *Eucommia ulmoides* Oliver using comparative transcriptome analyses. *Molecules*, *22*(12), p.2255.
79. Wu, X., Knapp, S., Stamp, A., Stammers, D.K., Jörnvall, H., Dellaporta, S.L. and Oppermann, U., 2007. Biochemical characterization of TASSELSEED 2, an essential plant short-chain dehydrogenase/reductase with broad spectrum activities. *The FEBS Journal*, *274*(5), pp.1172–1182.
80. Xu, H., Knox, R.B., Taylor, P.E. and Singh, M.B., 1995. Bcp1, a gene required for male fertility in Arabidopsis. *Proceedings of the National Academy of Sciences*, *92*(6), pp.2106–2110.
81. Xu, L., Hu, K., Zhang, Z., Guan, C., Chen, S., Hua, W., Li, J., Wen, J., Yi, B., Shen, J. and Ma, C., 2016. Genome-wide association study reveals the genetic architecture of flowering time in rapeseed (*Brassica napus* L.). *DNA Research*, *23*(1), pp.43–52.
82. Yang, H.W., Akagi, T., Kawakatsu, T. and Tao, R., 2019. Gene networks orchestrated by MeGI: a single-factor mechanism underlying sex determination in persimmon. *The Plant Journal*, *98*(1), pp.97–111.
83. Yang, Y., Tao, J. and Zong, S., 2020. Identification of putative Type-I sex pheromone biosynthesis-related genes expressed in the female pheromone gland of *Streltzoviella insularis*. *PloS One*, *15*(1), p.e0227666.
84. Yuan, Z. and Zhang, D., 2015. Roles of jasmonate signalling in plant inflorescence and flower development. *Current Opinion in Plant Biology*, *27*, pp.44–51.
85. Zarkower, D. and Hodgkin, J., 1993. Zinc fingers in sex determination: only one of the two *C. elegans* Tra-1 proteins binds DNA in vitro. *Nucleic acids research*, *21*(16), pp.3691–3698.
86. Zerpa-Catanho, D., Wai, J., Wang, M.L., Nguyen, J. and Ming, R., 2019. Differential gene expression among three sex types reveals a MALE STERILITY 1 (CpMS1) for sex differentiation in papaya. *BMC Plant Biology*, *19*(1), pp.1–22.
87. Zhou, R., Macaya-Sanz, D., Schmutz, J., Jenkins, J.W., Tuskan, G.A. and DiFazio, S.P., 2020. Sequencing and analysis of the sex determination region of *Populus trichocarpa*. *Genes*, *11*(8), p.843.
