# Supplementary material for: Genome-Wide Association Studies for Sex Determination and Cross-Compatibility in Water Yam (Dioscorea alata L.)
Source: Plants (Basel). 2021 Jul 10;10(7):1412. doi: 10.3390/plants10071412 (PMC8309230; doi:10.3390/plants10071412)
Supplement: Supplementary file 1 [file plants-10-01412-s001.zip › Supplementary Table S4.pdf]

Table S4. Soil and weather variables of the IITA yam breeding sites, Nigeria

**Parameters**	**Abuja**	**Ibadan**
Latitude	9°10′ N	7°29′ N
Longitude	7°21′ E	3°54′ E
Soil type	Sandy clay loam	Sandy loam
Soil pH	6.4	6.2
Average annual temperature, °C	26.0	25.9
Minimum annual temperature, °C	21.3	22.4
Maximum annual temperature, °C	32.9	31.6
Annual rainfall, mm	1363.9	1546.9
Rainy days per year	136.5	115.6
Evaporation, mm	-	1261.5
Minimum relative humidity, %	37.0	50.2
Maximum relative humidity, %	78.2	92.8
Sunshine, h	-	5.8
Solar radiation, MJ m^{-2} day^{-1}	16.6	15.5
Wind speed, m s^{-1}	1.7	3.1
Altitude, masl	~300	~220

Sources: IITA weather stations, Norman et al. (2018). Weather variables represent the mean of 11-year data record (2010–2020).

Norman, P.E.; Asfaw, A.; Tongoona, P.B.; Danquah, A.; Danquah, E.Y.; De Koeyer, D.; Asiedu, R. Pollination success in some white yam genotypes under polycross and nested mating designs. *Int. J. Biol. Sci. Appl.* **2018**, *5*, 19–28.
