# Supplementary material for: Genome-Wide Association Studies for Sex Determination and Cross-Compatibility in Water Yam (Dioscorea alata L.)
Source: Plants (Basel). 2021 Jul 10;10(7):1412. doi: 10.3390/plants10071412 (PMC8309230; doi:10.3390/plants10071412)
Supplement: Supplementary file 1 [file plants-10-01412-s001.zip › Supplementary Tables S1-2.pdf]

Table S1. Haplotype view of markers associated with plant sex in female plant of *D. alata*.

Markers	Variants	Homozygosity (%)	Heterozygosity (%)	Missing (%)
Chr11_27942	C/T	96.97	0.00	3.03
Chr6_112146	C/A	100.00	0.00	0.00
Chr6_120114	T/C	96.97	3.03	0.00
Chr6_135364	A/T	100.00	0.00	0.00
Chr6_135482	C/A	93.94	6.06	0.00
Chr6_136378	G/A	100.00	0.00	0.00
Chr6_140205	G/A	100.00	0.00	0.00
Chr6_140396	A/T	100.00	0.00	0.00
Chr6_141421	C/A	100.00	0.00	0.00
Chr6_14489	G/T	100.00	0.00	0.00
Chr6_1507	A/G	96.97	3.03	0.00
Chr6_15081	C/T	96.97	3.03	0.00
Chr6_1690	A/G	96.97	3.03	0.00
Chr6_1920	G/T	100.00	0.00	0.00
Chr6_19703	T/C	48.48	0.00	51.52
Chr6_2040	A/C	100.00	0.00	0.00
Chr6_20526	C/A	100.00	0.00	0.00
Chr6_20722	A/G	45.45	0.00	54.55
Chr6_20788	G/A	100.00	0.00	0.00
Chr6_20935	T/C	96.97	3.03	0.00
Chr6_21076	G/A	100.00	0.00	0.00
Chr6_25664	C/T	100.00	0.00	0.00
Chr6_29692	G/A	96.97	3.03	0.00
Chr6_3465	T/C	93.94	0.00	0.00
Chr6_3968	A/C	100.00	0.00	0.00
Chr6_4027	C/T	96.97	3.03	0.00
Chr6_41989	C/T	100.00	0.00	0.00
Chr6_44316	G/A	100.00	0.00	0.00
Chr6_44382	G/A	100.00	0.00	0.00
Chr6_4576	G/T	100.00	0.00	0.00
Chr6_4766	C/T	100.00	0.00	0.00
Chr6_4822	A/G	100.00	0.00	0.00
Chr6_48851	T/A	100.00	0.00	0.00
Chr6_48895	G/T	100.00	0.00	0.00
Chr6_53555	A/G	100.00	0.00	0.00
Chr6_53556	A/G	100.00	0.00	0.00
Chr6_53812	G/T	90.91	3.03	6.06
Chr6_5823	T/C	100.00	0.00	0.00
Chr6_58872	C/A	100.00	0.00	0.00
Chr6_60741	A/C	100.00	0.00	0.00
Chr6_60807	C/T	100.00	0.00	0.00
Chr6_659402	T/C	100.00	0.00	0.00
Chr6_66206	A/G	90.91	9.09	0.00
Chr6_70719	A/G	100.00	0.00	0.00
Chr6_74310	G/A	100.00	0.00	0.00

Chr6_745	T/A	100.00	0.00	0.00
Chr6_80861	G/A	48.48	0.00	51.52
Chr6_83712	T/C	100.00	0.00	0.00
Chr6_837364	T/C	96.97	3.03	0.00
Chr6_843525	C/A	96.97	3.03	0.00
Chr6_85928	T/C	96.97	0.00	3.03
Chr6_88389	G/A	100.00	0.00	0.00
Chr6_9161	T/A	100.00	0.00	0.00
Chr6_94183	C/T	100.00	0.00	0.00
Average percentage		95.90	0.84	3.14

Table S2. Haplotype view of markers associated with plant sex in male plant of *D. alata*.

Markers	Variants	Homozygosity (%)	Heterozygosity (%)	Missing (%)
Chr11_27942	C/T	7.32	90.24	2.44
Chr6_112146	C/A	17.07	82.93	0.00
Chr6_120114	T/C	14.63	85.37	0.00
Chr6_135364	A/T	9.76	90.24	0.00
Chr6_135482	C/A	2.44	97.56	0.00
Chr6_136378	G/A	26.83	73.17	0.00
Chr6_140205	G/A	14.63	85.37	0.00
Chr6_140396	A/T	4.88	95.12	0.00
Chr6_141421	C/A	4.88	95.12	0.00
Chr6_14489	G/T	14.63	82.93	0.00
Chr6_1507	A/G	2.44	97.56	2.44
Chr6_15081	C/T	2.44	97.56	0.00
Chr6_1690	A/G	4.88	95.12	0.00
Chr6_1920	G/T	2.44	97.56	0.00
Chr6_19703	T/C	75.61	24.39	0.00
Chr6_2040	A/C	4.88	95.12	0.00
Chr6_20526	C/A	2.44	97.56	0.00
Chr6_20722	A/G	82.93	17.07	0.00
Chr6_20788	G/A	26.83	73.17	0.00
Chr6_20935	T/C	48.78	51.22	0.00
Chr6_21076	G/A	2.44	97.56	0.00
Chr6_25664	C/T	51.22	48.78	0.00
Chr6_29692	G/A	2.44	97.56	0.00
Chr6_3465	T/C	53.66	46.34	0.00
Chr6_3968	A/C	2.44	97.56	0.00
Chr6_4027	C/T	2.44	97.56	0.00
Chr6_41989	C/T	2.44	97.56	0.00
Chr6_44316	G/A	2.44	97.56	0.00
Chr6_44382	G/A	2.44	97.56	0.00
Chr6_4576	G/T	2.44	97.56	0.00
Chr6_4766	C/T	2.44	97.56	0.00
Chr6_4822	A/G	2.44	97.56	0.00
Chr6_48851	T/A	2.44	97.56	0.00

Chr6_48895	G/T	2.44	97.56	0.00
Chr6_53555	A/G	24.39	75.61	0.00
Chr6_53556	A/G	31.71	68.29	0.00
Chr6_53812	G/T	53.66	46.34	0.00
Chr6_5823	T/C	2.44	97.56	0.00
Chr6_58872	C/A	7.32	97.56	0.00
Chr6_60741	A/C	7.32	97.56	0.00
Chr6_60807	C/T	7.32	97.56	0.00
Chr6_659402	T/C	7.32	92.68	0.00
Chr6_66206	A/G	7.32	97.56	0.00
Chr6_70719	A/G	7.32	97.56	0.00
Chr6_74310	G/A	2.44	97.56	0.00
Chr6_745	T/A	2.44	97.56	0.00
Chr6_81373	G/A	24.39	75.61	0.00
Chr6_83712	T/C	2.44	97.56	0.00
Chr6_837364	T/C	56.10	43.90	0.00
Chr6_843525	C/A	56.10	43.90	0.00
Chr6_85928	T/C	7.32	92.68	0.00
Chr6_88389	G/A	2.44	97.56	0.00
Chr6_9161	T/A	12.20	85.37	2.44
Chr6_94183	C/T	2.44	97.56	0.00
Average percentage		15.36	84.96	0.14
